# Using an Endovascular Strategy in the Open Setting: The Challenges of Hybrid Repair Using Frozen Elephant Trunk in an Extensively Dilated Aorta Associated with Coarctation

**DOI:** 10.1055/s-0041-1732398

**Published:** 2021-12-08

**Authors:** Bertram Harrington, Iain McPherson, Christopher Bayliss, Sion Barnard, James McCaslin, Robin Williams, Karen Booth

**Affiliations:** 1 Newcastle University Medical School, Framlington Place, Newcastle-upon-Tyne, United Kingdom; 2 Department of Cardiothoracic Surgery, Freeman Hospital, Newcastle-upon-Tyne, United Kingdom

**Keywords:** aortic arch, hybrid intervention, frozen elephant trunk, coarctation of the aorta, aneurysm

## Abstract

With both stenosis and aneurysm, repairing a severely tortuous and coarcted aorta can present certain difficulties. The advent of hybrid arch frozen elephant trunk techniques, as well as other endovascular solutions, has produced safer surgical repair methods for such cases. We present the reconstruction and repair of a Type-1 thoracoabdominal aortic aneurysm using a staged approach in less-than-optimal anatomy. Interventions included hybrid frozen elephant trunk, balloon dilation, and thoracic endovascular repair.

## Introduction

Coarctation of the aorta has an incidence of 0.3 per 1,000 population and is often not discovered until adulthood. When untreated, it can lead to congestive heart failure. 1 It is linked to many differing cardiac pathologies including bicuspid aortic valve disease, aortic root dilatation, and poststenotic thoracic aortic dilatation. 2 3 Traditionally, a staged approach was required to treat the aortic root followed by the descending thoracic aorta. Each procedure has a relatively low mortality and morbidity risk with the added risk of rupture of the untreated aorta while recovering from the first procedure. 4 5 Frozen elephant trunk (FET) aims to treat all portions of the aorta in a single procedure or allows for a hybrid approach by providing a landing zone for subsequent endovascular intervention. We present this hybrid approach and discuss the technical challenges associated with it.

## Case Presentation

**Fig. 1:**
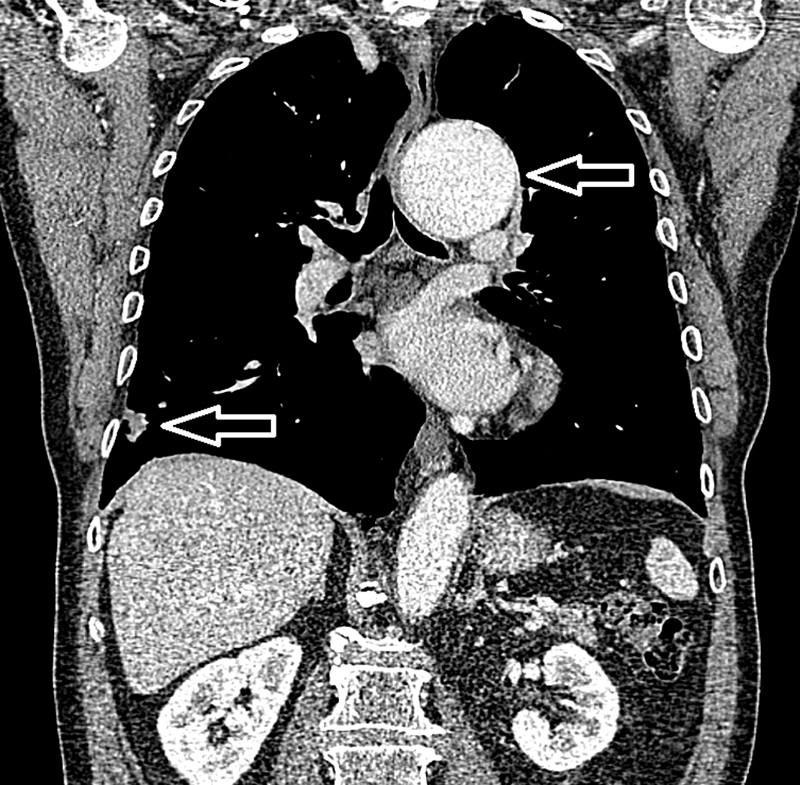
Preoperative computed tomography (CT) demonstrating proximal descending aorta dilation (top arrow) and right lower lobe lesion (bottom arrow). It was identified in the preoperative CT that the patient had both a dilated aortic root and postcoarctation dilation extending to the proximal descending aorta.

A 78-year-old male, former smoker, was referred to cardiothoracic services after investigation for a 12-month history of worsening dyspnea, dry cough, and long-term hoarse voice. Computed tomography (CT) of the thorax revealed a spiculated right lower lobe lesion and incidental finding of aortic aneurysm ( Fig. 1 ). A CT-guided biopsy of the lesion was attempted but was unsuccessful.

CT of the aorta ( Fig. 1 ) demonstrated a 62-mm dilated aortic root tapering toward a normal caliber arch with further Type-1 thoracoabdominal aneurysm related to postcoarctation stenotic dilatation and severe tortuosity. This coarctation had not resulted in any specific clinical consequences and was solely an incidental finding. Preoperative transthoracic echocardiogram displayed a moderately stenosed calcified bicuspid aortic valve (peak gradient: 41.22 mm Hg, valve area: 1.13 cm ^2^ ) with a trace of regurgitation. Left ventricular function was mildly impaired with an ejection fraction of 50 to 55%. The patient was reviewed by both cardiac and thoracic surgical teams. After discussion, the decision was made to approach both pathologies in one procedure as part of a hybrid strategy, performing the root, ascending and arch replacement using a FET alongside a diagnostic wedge resection of the right lower lobe.

**Fig. 2:**
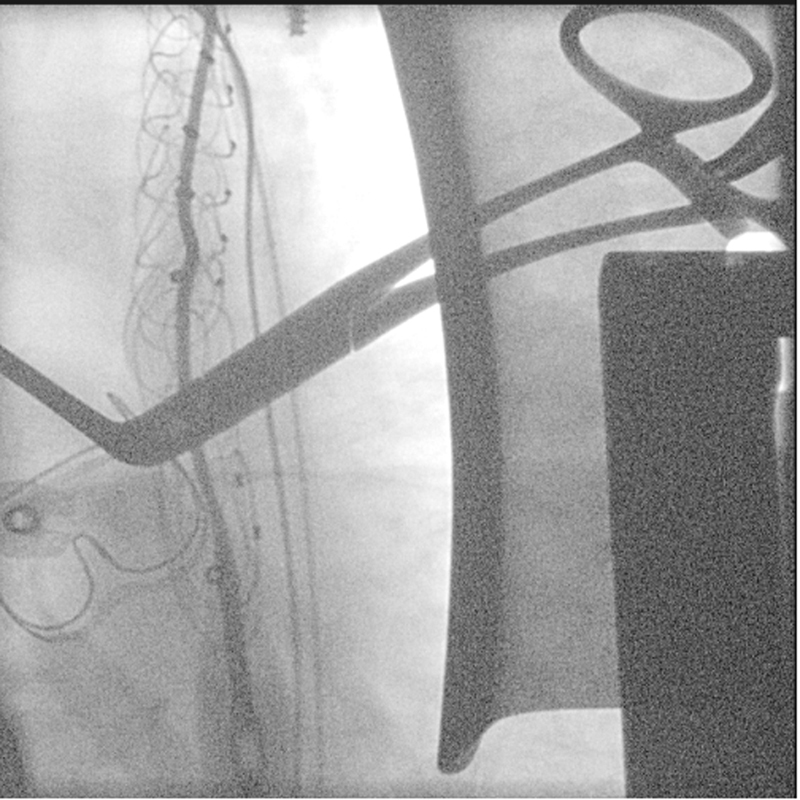
Intraoperative fluoroscopy showing positioning of the elephant trunk stent using two wires. A two-wire approach was employed to overcome the technically challenging anatomy.

Routine median sternotomy was performed, and cardiopulmonary bypass established via two-stage venous cannulation with aortic return and a right superior pulmonary vein vent. A modified Bentall procedure was performed without complication using a 30-mm Terumo aortic graft and 27-mm INSPIRIS RESILIA Aortic Valve (Edwards Lifesciences, One EdwardsWay, Irvine, California) pericardial aortic valve, constructed using continuous 4–0 prolene. The patient was cooled to a minimum of 18°C prior to circulatory arrest and cross-clamp removal. The aortic arch was opened, and cerebral perfusion established bilaterally with a mean arterial pressure target of 55 mm Hg (flow: 400–500 mL/minute) including occlusion of the left subclavian. Passage of the FET was challenging, given the extreme tortuosity and flaccidity of the aorta and narrowing. It was not initially possible to advance the delivery system of the Thoraflex FET; therefore, a double wire technique was used to advance the FET to the mid-descending thoracic aorta ( Fig. 2 ). The left subclavian artery was then anastomosed to the graft and the cuff of the elephant trunk completed (zone 2). Lower body perfusion was re-established after 60 minutes, the left common carotid artery and innominate artery anastomosed respectively, and the patient rewarmed. Total cross-clamp time was 305 minutes and the patient was successfully weaned from bypass on minimal inotropic support after a total time of 366 minutes.

**Fig. 3:**
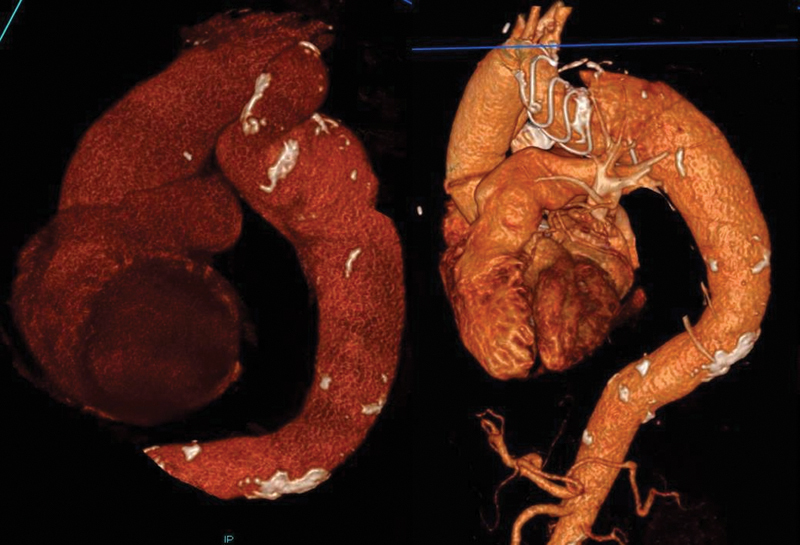
Pre- and postoperative three-dimensional rendered computed tomography imaging demonstrating severe tortuosity and the partial graft collapse. Partial graft collapse was corrected in a follow-up procedure along with the deployment of thoracic endovascular aortic repair.

A pleural incision was performed after normalization of the activated clotting time to permit a right lower lobe stapled wedge resection. This was sent for histopathological analysis and was reported as showing pulmonary nodular amyloidosis and fibrosis with no malignancy. The patient went on to have an uneventful postoperative recovery. The postoperative CT ( Fig. 3 ) demonstrates the clinical challenges we faced with the foreshortening evident in the nitinol frame. The patient was asymptomatic as before intervention and went on to receive second-stage intervention of thoracic endovascular aortic repair and balloon dilatation on postoperative day 10 to the FET. This was to fully exclude the thoracic aneurysm and complete correction of the coarctation. These procedures are routinely staged at our unit based on previous experience and the patient's recovery between interventions.

## Discussion

We read with interest the case reports in the literature on this clinical scenario and, in particular, the comments of Gaudino 6 with regard to “being smart over being brave.” Hybrid approaches allow surgeons to treat clinically complex problems with curative intent at acceptable risk to the patient. In this case, our concern was that full deployment of the FET with a good seal in such challenging anatomy would not be possible.

Endovascular solutions are known to demonstrate a degree of safety in the short term and have the potential to be more cost effective than two-stage open repair. They do, however, come with the added risks of late-term failure, as they are subject to displacement, endoleak, or graft failure. 7 8 In our minds, the option of two-stage open surgery would be preferred in a younger age group. We feel, this hybrid approach has been reproducible and, as only three case reports are available in the literature, is worthy of sharing and discussion.
